# Technical Determinants of On-Water Rowing Performance

**DOI:** 10.3389/fspor.2020.589013

**Published:** 2020-12-03

**Authors:** Ana C. Holt, Robert J. Aughey, Kevin Ball, William G. Hopkins, Rodney Siegel

**Affiliations:** ^1^Institute for Health and Sport (IHeS), Victoria University, Melbourne, VIC, Australia; ^2^Sport Science Department, Victorian Institute of Sport, Melbourne, VIC, Australia; ^3^Australian Institute of Sport, Canberra, ACT, Australia

**Keywords:** technique, force curve, oar angle, race, stroke rate, power output, individual differences

## Abstract

**Purpose:** Research establishing relationships between measures of rowing technique and velocity is limited. In this study, measures of technique and their effect on rowing velocity were investigated.

**Methods:** Ten male singles, eight female singles, three male pairs, and six female pairs participated. Data from each stroke for forty-seven 2,000 m races were collected using Peach PowerLine and OptimEye S5 GPS units. General linear mixed modeling established modifying effects on velocity of two within-crew SD of predictor variables for each boat class, with subsequent adjustment for power, and for power and stroke rate in separate analyses. Twenty-two predictor variables were analyzed, including measures of boat velocity, gate force, and gate angle. Results were interpreted using superiority and inferiority testing with a smallest important change in velocity of 0.3%.

**Results:** Substantial relationships with velocity were found between most variables assessed before adjustment for power, and for power and stroke rate. Effect magnitudes were reduced for most variables after adjustment for power and further reduced after adjustment for stroke rate and power, with precision becoming inadequate in many effects. The greatest modifying effects were found for stroke rate, mean and peak force, and power output before adjustment, and for catch angle after adjustment for stroke rate and power. Substantial between-crew differences in effects were evident for most predictors in some boat classes before adjustment and in some predictors and some boat classes after adjustment for stroke rate and power.

**Conclusion:** The results presented reveal variables associated with improvements in rowing performance and can be used to guide technical analysis and feedback by practitioners. Higher stroke rates and greater catch angles should be targeted to improve rowing performance, and rower force development for the improvement of power output. Relationships between rowing technique and velocity can be crew-dependent and are best assessed on an individual basis for some variables.

## Introduction

Rowing is a sport with high technical demand, whereby an athlete's on-water performance ability is a product of not only their physiological work capacity but also their technical ability. With the use of instrumentation systems, practitioners have the means for quantitative assessment of multiple variables associated with rowing technique, allowing feedback to coaches and athletes regarding beneficial areas of technical focus. Key areas for technical assessment include the oar angle rowed through (arc angle) and the application of force to the oar, as these measures contribute to propulsive work (Warmenhoven et al., 2018). Longer arc angles achieved through more negative catch and more positive finish angles as well as smaller catch and finish slips are often sought by coaches. However, contradictory findings regarding the direction of the relationships between these variables and boat velocity have been observed (Coker, 2010). The effect of oar angle achievement on velocity has not been compared between boat classes and genders, although larger arc angles are achievable in sculling (two oars per rower) than sweep rowing (one oar per rower), and smaller arc angles have been reported in females compared to males (Kleshnev et al., 1998; Coker, 2010). Arc angle is also related to stroke rate, as arc angles decrease through reductions occurring predominantly to catch angle with increases in stroke rate above 24 strokes·min^−1^ (Kleshnev, 2007).

Power output is commonly assessed by practitioners and has moderate to large relationships with velocity in scullers (Coker, 2010). Strong positive relationships also exist between power and stroke rate (Hofmijster et al., 2007; Held et al., 2019). The rate of force development and the occurrence of peak force at more negative oar angles are associated with more successful scullers (Warmenhoven et al., 2017). Larger mean-to-peak force ratios (representing more consistent force application) exist in elite men's pairs compared to sub-elite pairs (Smith and Draper, 2006). Similarly, higher peak and mean forces, with more negative peak force oar angles, have been reported for senior compared to underage rowers (Kleshnev et al., 1998), likely related to increased force development capacity with age. However, how these relationships differ between boat classes and genders has not yet been examined.

Research investigating relationships between measures of rowing technique and performance is valuable for informing technical analysis and athlete feedback. However, further investigation to determine the direction of relationships with boat velocity where findings are contradictory, and to explore differences between boat classes and genders in relationships between technical measures and velocity, is needed, as these are rarely compared. Furthermore, the investigation of associations between additional measures of rowing technique and boat velocity that have not yet been assessed is warranted. Therefore, the purpose of this study was to investigate the separate effects of multiple measures of rowing technique on boat velocity in men's and women's singles and coxless pairs during 2,000 m racing. The outcomes of this study will advise which technical measures have the greatest associations with rowing performance, informing practitioner assessment and feedback regarding rowing technique.

## Methods

### Subjects

Seventeen female (age 20.7 ± 2.4 years; height 177.2 ± 6.3 cm; body mass 73.6 ± 7.8 kg) and 14 male (age 21.6 ± 2.8 years; height 189.3 ± 8.1 cm; body mass 84.8 ± 10.4 kg) national-pathway rowers who performed regular training volumes of ~17–22 h·week^−1^ volunteered for this study. Participants provided informed consent prior to commencement of the study. The study was approved by the university ethics committee and conforms to the Code of Ethics of the World Medical Association.

### Methodology

The study was conducted during two national regattas held at the Sydney International Rowing Center. A total of forty-seven 2,000 m races were recorded from 10 male single scull crews (17 races), eight female single scull crews (13 races), three male coxless pair crews (five races), and six female coxless pair crews (12 races). Crew age categories were <21 years (three crews), <23 years (18 crews), and senior (>22 years; five crews). Races recorded were heats (20 races), repechages (three races), semi-finals (three races), and finals (21 races). Crews were given no instructions from the researchers regarding race strategy or stroke rate. Power output and predictor variables were collected per stroke from races using Peach PowerLine instrumentation systems (Peach Innovations, UK) with a 50 Hz sample rate. Boat velocity and acceleration were collected with a 10 Hz sample rate from OptimEye S5 GPS units (Catapult, Australia) attached to participant boats. Both Peach PowerLine instrumentation and Catapult GPS systems are used frequently within elite rowing programs. Acceptable levels of validity have been established for measures of rowing velocity from Catapult GPS units (Smith and Hopkins, 2012) and for force and oar angle by Peach instrumentation systems (Coker et al., 2009). Power provided by the Peach instrumentation system represents a proxy measure of the true mechanical power output (Hofmijster et al., 2018). Venue environmental conditions (collected at 1 min intervals from six weather stations positioned at water level along the 2,000 m course) were: 22.8 ± 2.1°C air temperature (mean ± *SD*), 26.0 ± 1.3°C water temperature, 59.0 ± 10.3% relative humidity, and 1.4 ± 0.6 m·s^−1^ wind speed, in a predominantly cross-tail direction on stroke side.

The predictor variables assessed were measures available from the combination of Peach and GPS data, and most were common variables used by practitioners and coaches in technical analysis. Peach and OptimEye S5 race data were combined using the software Logan (version 48.41, Catapult, Australia) to align acceleration traces from each device. Strokes were partitioned from catch to catch, with the catch identified as the largest negative gate angle, the finish as the largest positive gate angle achieved during one stroke cycle, and arc angle as the absolute difference in gate angle between catch and finish angles per stroke. The drive was defined as the period between the catch and the subsequent finish, and the recovery as the period between the finish and the catch of the subsequent stroke. Most predictor variables presented in Table 1 were calculated per stroke using Logan and exported for statistical analysis. Power output [W; as measured by Peach from gate angle velocity, gate force in the direction of the boat's long axis, and the ratio of the oar outboard (distance from the collar to blade tip) to total length], peak force angle (°; the gate angle during the drive where peak force occurs), and catch and finish slip per stroke were exported from PowerLine (version 4.02, Peach Innovations, UK). Catch and finish slips were defined as the gate angles rowed through at the catch and finish respectively, where gate force was below the standard force thresholds of 196 N (catch slip) and 98 N (finish slip) (Coker, 2010). Predictor variables exported from Logan include stroke rate (stroke·min^−1^), within-stroke velocity range (m·s^−1^; difference between maximum and minimum boat velocities occurring within each stroke), time from catch to minimum velocity (s; duration from catch to minimum boat velocity occurring in the early drive), distance per stroke (m), mean force (N; mean force measured at the gate during the drive), peak force (N; highest force measured at the gate during the drive), rate of force development (N·s^−1^; change in force over change in time between the catch and peak force occurrence during the early-to-mid drive), time to peak force from the catch (s; duration from the catch to the occurrence of peak force during the drive), and mean-to-peak force ratio (calculated as peak force divided by mean force). The first 10 strokes of each race were excluded from analyses to remove outliers, with the remaining strokes from each race assessed.

**Table 1:** Characteristics of the predictor variables in the four boat classes. Data are mean ± between-crew SD/within-crew SD^a^.

	**Single sculls**		**Coxless pairs**	
	**Men (M1x)**	**Women (W1x)**	**Men (M2-)**	**Women (W2-)**
**Time and boat-velocity variables**				
Stroke rate (strokes·min^−1^)	34.7 ± 1.7/2.0	32.8 ± 1.0/1.8	38.1 ± 0.7/1.7	35.1 ± 2.0/2.3
Within-stroke velocity range (m·s^−1^)	2.27 ± 0.20/0.13	2.14 ± 0.12/0.12	2.71 ± 0.11/0.12	2.30 ± 0.12/0.14
Time from catch to minimum velocity (s)	0.14 ± 0.03/0.04	0.12 ± 0.04/0.02	0.13 ± 0.00/0.01	0.15 ± 0.01/0.03
Distance per stroke (m)	7.97 ± 0.43/0.33	7.65 ± 0.18/0.29	7.82 ± 0.19/0.17	7.38 ± 0.34/0.28
**Gate force variables**				
Mean force (N)	261 ± 26/21	199 ± 17/16	503 ± 40/40	367 ± 34/38
Power output (W)	334 ± 33/34	223 ± 21/25	760 ± 38/92	481 ± 43/58
Peak force (N)	497 ± 68/34	371 ± 48/30	968 ± 81/65	694 ± 97/68
Rate of force development (N·s^−1^)	960 ± 190/100	760 ± 270/190	1980 ± 240/190	1450 ± 270/200
Time to peak force from the catch (s)	0.43 ± 0.07/0.04	0.39 ± 0.04/0.04	0.36 ± 0.03/0.03	0.36 ± 0.07/0.05
Mean to peak force ratio	1.90 ± 0.13/0.08	1.87 ± 0.13/0.06	1.88 ± 0.16/0.06	1.89 ± 0.13/0.08
Peak force angle (°)	−20.1 ± 5.2/4.0	−28.5 ± 6.4/4.3	−14.5 ± 3.1/3.0	−18.5 ± 4.2/2.9
**Gate angle variables**				
Catch slip (°)	7.7 ± 3.0/1.8	9.7 ± 2.8/1.8	3.7 ± 3.2/1.2	5.6 ± 3.6/2.3
Finish slip (°)	14.1 ± 3.0/1.6	18.1 ± 3.5/2.1	8.5 ± 2.9/1.0	8.5 ± 2.0/1.5
Finish angle (°)	43.5 ± 2.3/1.4	44.2 ± 3.1/1.3	33.2 ± 1.1/1.4	32.4 ± 1.7/1.2
Arc angle (°)	105.4 ± 5.2/2.5	106.0 ± 3.2/2.2	82.0 ± 2.3/1.2	80.4 ± 3.5/2.8
Catch angle (°)	−62.0 ± 5.2/1.8	−61.8 ± 2.3/1.3	−48.8 ± 2.8/1.7	−48 ± 3.8/2.0

*M1x, men's single scull; W1x, women's single scull; M2-, men's coxless pairs; W2- women's coxless pairs*.

a *Mean is the mean of the crew means, between-crew SD is the SD of the crew means, and within-crew SD is the mean of the crews' SDs across their one to the three races (~250 to ~750 strokes)*.

*Number of crews: 10, 8, 3, and 6 respectively*.

*Number of races: 17, 13, 5, and 12 respectively*.

### Statistical Analysis

Each gender and boat class was analyzed separately with the general linear mixed-model procedure (Proc Mixed) in the Studio University edition of the Statistical Analysis System (version 9.4, SAS Institute, Cary NC). The fixed effects in the model, predicting the logarithm of boat velocity (V), were each predictor variable presented in Table 1 analyzed separately as linear predictors, allowing estimation of the mean modifying effect on velocity of two within-crew standard deviations (one above and one below the adjusted mean of zero). Separate analyses were conducted to adjust for power output and to adjust for power output and stroke rate; in these analyses, log(V) was predicted by the logarithm of the sum of the mean stroke power (P) of both oars, allowing estimation of k and x in the kinetic equation V = k·P^x^, where the exponent x was allowed to vary between crews, to adjust for individual differences in its value. Fixed effects in the model were log(P) and log(stroke rate) [adjusting log(V) for power output and stroke rate] and each predictor variable separately as linear predictors. Effects on boat velocity were assessed over a two-standard-deviation (SD) within-crew change in each predictor (Hopkins et al., 2009). The modifying effects of gate force and gate angle variables on velocity were estimated for the bow and stroke side; similar effects were generally observed for both sides, so the summed effect of the sides is presented for force measures, and the mean effect of the sides is presented for angle and time measures.

Random effects in the model were: crew identity (to adjust for consistently better or worse velocity of each crew across all races); the given predictor variable (for bow and stroke sides, where relevant) interacted with crew identity (to estimate individual differences between crews in the effect of the variable); race identity (to adjust for between-race changes in mean velocity due to changes in environmental conditions and the efficiency of the crew); and a different residual error for each crew (representing stroke-to-stroke variability in velocity not accounted for by the other effects). The random effects for stroke- and bow-side variables were combined by summing their variances for force measures, and taking their mean variance for angle and time measures, on the assumption that they acted independently. The random effects for crew identity and race identity represent adjustments to improve precision and therefore do not contribute directly to the effects of technique variables on boat velocity; these effects will be reported elsewhere.

A smallest important change in velocity of 0.3% was assumed, given the 1.0% race-to-race variation in 2,000 m race times of elite rowers (Smith and Hopkins, 2011). Corresponding magnitude thresholds for changes in velocity were: <0.3% trivial, ≥0.3% small, ≥0.9% moderate, ≥1.6% large, ≥2.5% very large, and ≥4.1% extremely large (Hopkins et al., 2009). To evaluate magnitudes of standard deviations representing between-crew differences in the modifying effect of each predictor variable on boat velocity, the square of the standard deviation was assumed to be normally distributed, and the magnitude thresholds are one-half of those in the above scales, equivalent to evaluating two standard deviations with the above thresholds: <0.15% trivial, ≥0.15% small, ≥0.45% moderate, ≥0.8% large, ≥1.3% very large, and ≥2.0% extremely large (Smith and Hopkins, 2011).

Sampling uncertainty in the estimates of effects is presented as 90% compatibility limits. Decisions about magnitudes accounting for the uncertainty were based on one-sided interval hypothesis tests, according to which a hypothesis of a given magnitude (substantial, non-substantial) is rejected if the 90% compatibility interval falls outside that magnitude (Aisbett et al., 2020; Hopkins, 2020). *P*-values for the tests were therefore the areas of the sampling distribution of the effect (*t* for means, *z* for variances) falling within the hypothesized magnitude, with the distribution centered on the observed effect. Hypotheses of inferiority (substantial-negative) and superiority (substantial-positive) were rejected if their respective p values (p_−_ and p_+_) were <0.05; rejection of both hypotheses represents a decisively trivial effect in equivalence testing. When only one hypothesis was rejected, the p value for the other hypothesis, when >0.25, was interpreted as the posterior probability of a substantial true magnitude of the effect in a reference-Bayesian analysis with a minimally informative prior (Hopkins et al., 2019) using the following scale: >0.25, possibly; >0.75, likely; >0.95, very likely; and >0.995, most likely (Hopkins et al., 2009). The probability of a trivial true magnitude (1 – p_−_ – p_+_) was also interpreted, when >0.25, with the same scale, which should help researchers and practitioners to understand the uncertainty in the effects. Probabilities were not interpreted for effects with inadequate precision at the 90% level, defined by failure to reject both hypotheses (*p*_−_ > 0.05 and *p*_+_ > 0.05). Effects with adequate precision at the 99% level (*p*_−_ < 0.005 or *p*_+_ < 0.005) are shown in bold in the **Supplementary Tables**, since these represent effects that have a conservative low risk of harm (most unlikely to impair performance), if implemented. The hypothesis of non-inferiority (non-substantial-negative) or non-superiority (non-substantial-positive) was rejected if its *p*-value (p_N−_ = 1 – p_−_ or p_N+_ = 1 – p_+_) was <0.05, representing a decisively substantial effect in minimal-effects testing: very likely or most likely substantial.

## Results

**Figure 1:**
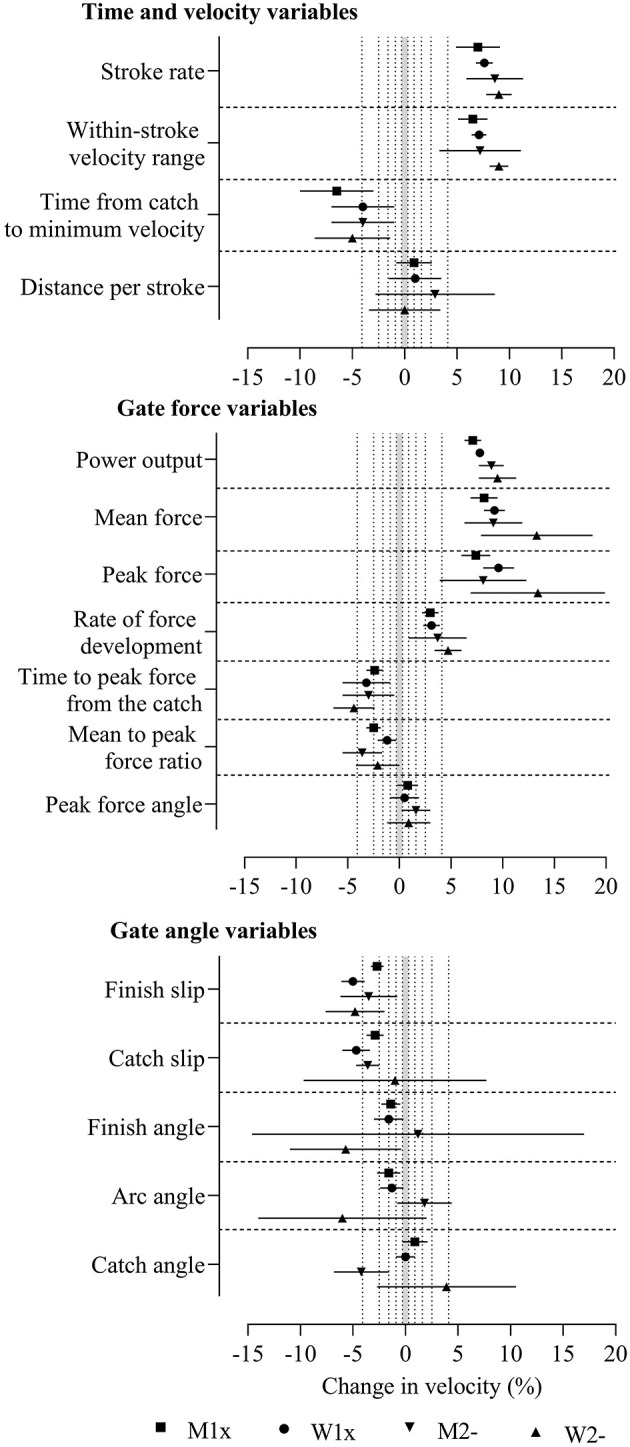
Change in boat velocity for a change in each predictor variable of two within-crew standard deviations in the four boat classes without adjustment. Data are mean (%) and 90% compatibility limits. The shaded grey area covers values within the smallest important change thresholds (-0.3% to 0.3%). Vertical dotted lines delineate threshold magnitudes of small (±0.3), moderate (±0.9), large (±1.6), very large (±2.5), and extremely large (±4.1). Rejection of the non-inferiority or non-superiority hypothesis occurs when compatibility limits do not enter the grey area. Effects with compatibility limits that end within the grey area have adequate precision but are only possibly or likely substantial. M1x, men's single sculls; W1x, women's singles sculls; M2-, men's coxless pairs; W2-, women's coxless pairs.

Mean values for predictor variables with between-crew and within-crew SD are presented in Table 1. Within-crew SD indicates half of the range that effects for predictors were assessed over. The mean modifying effects of predictor variables on boat velocity are presented in Figure 1 for all four boat classes and in Supplementary Table 1 with *p*_−_ and *p*_+_ values and reference-Bayesian likelihoods of substantial and trivial effects.

The greatest effects on velocity before adjustment were found for peak and mean force, which were extremely large, positive effects, where the non-superiority hypothesis was rejected in all boat classes (*p*_N+_ ≤ 0.003). Before adjustment, most effects were very large to extremely large and had sufficient precision for the true magnitudes to be very likely or most likely substantial (rejection of the non-superiority or non-inferiority hypotheses, p_N+_ or p_N−_ <0.05 or <0.005). Consistent positive effects in all boat classes were found for stroke rate, within-stroke velocity range, power output, peak and mean power, and rate of force development. Consistent negative effects in all boat classes were found for time from the catch to minimum velocity, time to peak force from the catch, and finish slip. Precision was inadequate (the non-superiority or non-inferiority hypothesis was not rejected, p_N+_ or p_N−_ > 0.05) in most boat classes for distance per stroke, peak force angle, and catch angle.

**Figure 2:**
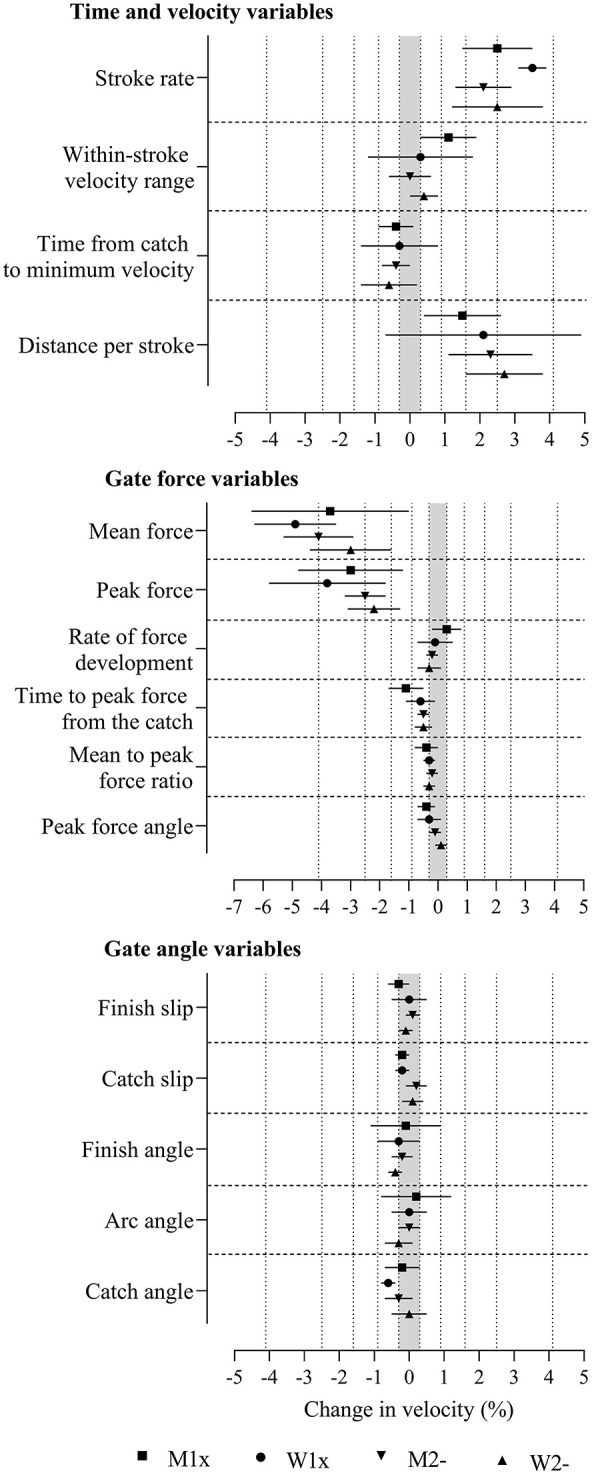
Change in boat velocity for a change in predictor variables of two within-crew standard deviations with adjustment for power output in the four boat classes. Data are mean (%) and 90% compatibility limits. The shaded grey area covers values within the smallest important change thresholds (-0.3% to 0.3%). Vertical dotted lines delineate threshold magnitudes of small (±0.3), moderate (±0.9), large (±1.6), very large (±2.5), and extremely large (±4.1). Rejection of the non-inferiority or non-superiority hypothesis occurs when compatibility limits do not enter the grey area. Effects with compatibility limits that end within the grey area have adequate precision but are only possibly or likely substantial. M1x, men's single sculls; W1x, women's singles sculls; M2-, men's coxless pairs; W2-, women's coxless pairs.

After the adjustment for power, effect magnitudes were reduced (Figure 2 and Supplementary Table 2). Large to very large positive effects were found in most boat classes for stroke rate and distance per stroke, large to extremely large negative effects across boat classes were found for mean and peak force, and small negative effects for time to peak force from the catch were found in some boat classes where precision was sufficient for the true magnitudes of these effects to be very likely or most likely substantial (rejection of the non-substantial hypotheses, p_N+_ or p_N−_ <0.05 or <0.005). Measures that were potentially useful for practitioners, where the effects had adequate precision but were only possibly or likely substantial (one of the non-superiority or non-inferiority hypotheses was not rejected, p_N+_ or p_N−_ >0.05), include positive effects in some boat classes for within-stroke velocity range and rate of force development and negative effects for time from the catch to minimum velocity, time to peak force from the catch, mean-to-peak force ratio, peak force angle, finish slip, arc angle, finish angle, and catch angle. Less potentially useful were observed trivial effects that had adequate precision, where the true magnitudes were possibly or likely trivial but where only one of the superiority and inferiority hypotheses was rejected (p_N+_ or p_N−_ <0.05), found in some boat classes for rate of force development, mean-to-peak force ratio, peak force angle, catch and finish slips, arc angle, and finish angle. Only one effect was decisively trivial, where both the superiority and inferiority hypotheses were rejected (p_N+_ or p_N−_ <0.05), in women's singles for peak force angle.

**Figure 3:**
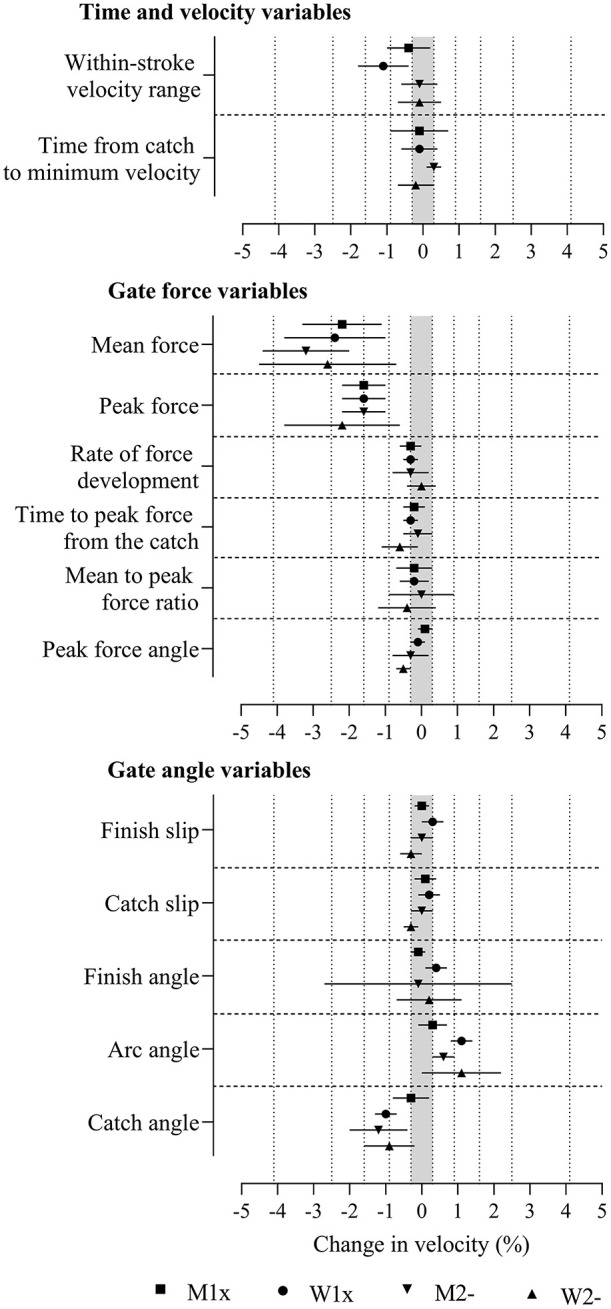
Change in boat velocity for a change in predictor variables of two within-crew standard deviations with adjustment for power output and stroke rate in the four boat classes. Data are mean (%) and 90% compatibility limits. The shaded grey area covers values within the smallest important change thresholds (-0.3% to 0.3%). Vertical dotted lines delineate threshold magnitudes of small (±0.3), moderate (±0.9), large (±1.6), very large (±2.5), and extremely large (±4.1). Rejection of the non-inferiority or non-superiority hypothesis occurs when compatibility limits do not enter the grey area. Effects with compatibility limits that end within the grey area have adequate precision but are only possibly or likely substantial. M1x, men's single sculls; W1x, women's singles sculls; M2-, men's coxless pairs; W2-, women's coxless pairs.

The adjustment of power and stroke rate further reduced the magnitudes of most effects to the ranges of trivial to large (Figure 3 and Supplementary Table 3). Distance per stroke is not presented in Figure 3 or Supplementary Table 3, because when multiplied by stroke rate (added, after log transformation), it perfectly predicts velocity. Moderate and small positive effects were found in some boat classes for arc angle, large to very large negative effects were found across boat classes for mean and peak force, and moderate negative effects were found in some boat classes for within-stroke velocity range and catch angle where precision was sufficient for the true magnitudes of these effects to be very likely or most likely substantial (rejection of the non-substantial hypotheses, p_N+_ or p_N−_ <0.05 or <0.005). Measures that were potentially useful for practitioners, where the effects had adequate precision but were only possibly or likely substantial (one of the non-superiority or non-inferiority hypotheses was not rejected, p_N+_ or p_N−_ >0.05) after adjustment for power and stroke rate, include positive effects in some boat classes for time from the catch to minimum velocity, finish slip, finish angle, and arc angle and negative effects for within-stroke velocity range, rate of force development, time to peak force from the catch, peak force angle, catch slip, and catch angle. Decisively trivial effects where both the superiority and inferiority hypotheses were rejected (p_N+_ or p_N−_ <0.05) were found for peak force angle and finish slip in men's singles. Trivial effects that had adequate precision, where the true magnitudes were possibly or likely trivial but where only one of the superiority and inferiority hypotheses was rejected (p_N+_ or p_N−_ <0.05), were found in some boat classes for time to peak force from the catch, mean-to-peak force ratio, peak force angle, catch slip, and finish angle.

Random error arising from the Peach or Catapult system attenuates the effect of the variable on boat velocity. Random errors estimated in a forthcoming study were 0.4, 0.3, and 0.5° for catch, finish, and arc angles, respectively. The attenuation, expressed as a modifying factor (Hopkins et al., 2009), was estimated using (SD^2^-e^2^)/SD^2^, where SD is the within-crew standard deviation (~1.7, ~1.3, and ~2.2°; Table 1) and e is the random error. The resulting factors were 0.94, 0.95, and 0.93, which are practically negligible. The extent to which random error modifies the effects of the other predictor variables is unknown.

Differences between crews in the effect of predictor variables on velocity before adjustment are presented in Supplementary Table 4 with p_−_ and p_+_ values and reference-Bayesian likelihoods of substantial and trivial effects. Between-crew differences in men's and women's singles were mostly large to extremely large and had adequate precision for the true magnitudes to be very likely or most likely substantial (rejection of the non-superiority or non-inferiority hypotheses, p_N+_ or p_N−_ <0.05 or <0.005). The magnitudes of between-crew differences were similar in pairs to those in singles, but precision was inadequate (rejection of one of the non-superiority or non-inferiority hypotheses, p_N+_ or p_N−_ <0.05) for some predictors in women's pairs and for most predictors in men's pairs (owing to an insufficient number of crews and races).

**Figure 4:**
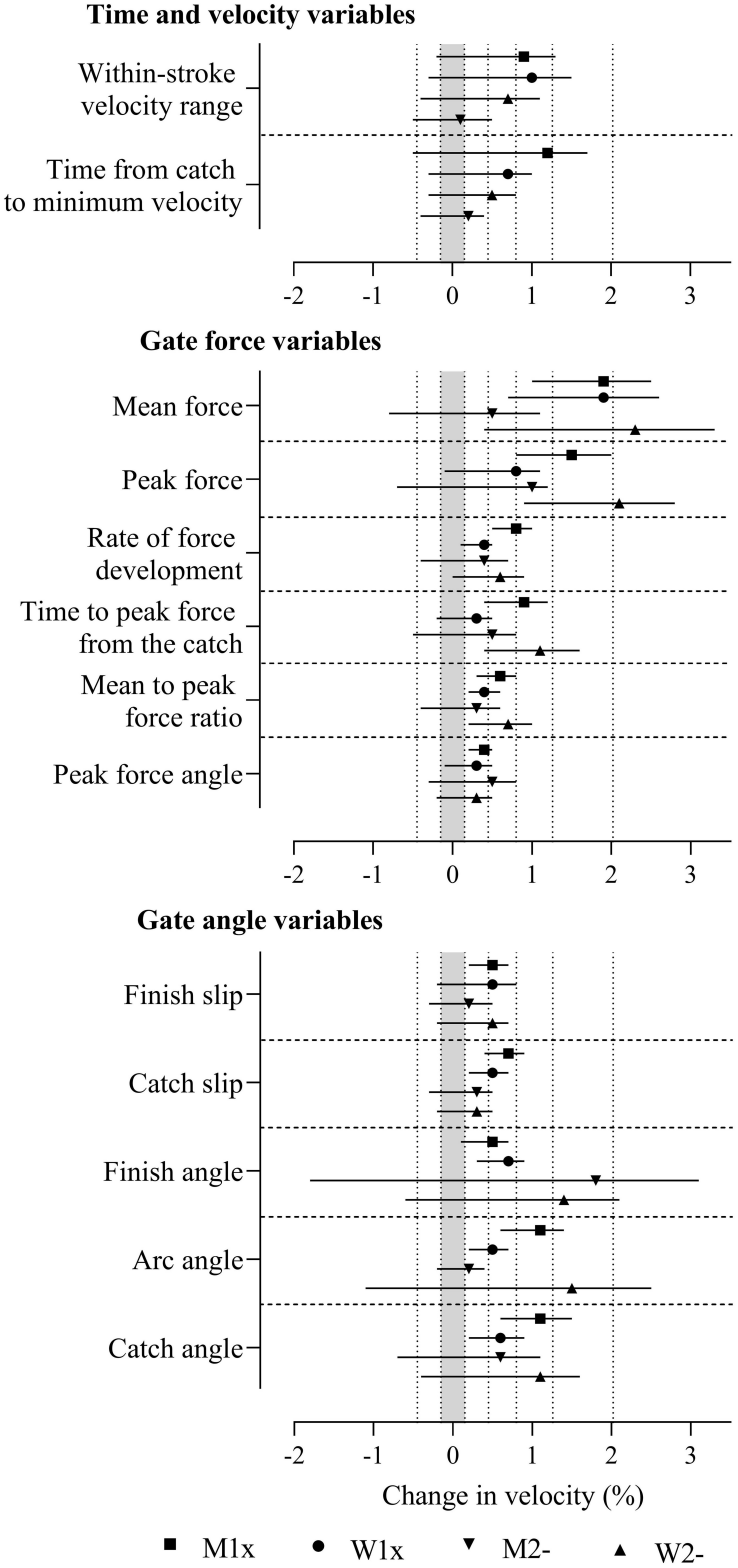
Differences between crews in the effects of the predictor variables shown in Figure 3 in the four boat classes with adjustment for power output and stroke rate. Data are SD (%) and 90% compatibility limits. The shaded grey area covers values within the smallest important difference thresholds (-0.15% to 0.15%). Vertical dotted lines delineate threshold magnitudes of small (±0.15), moderate (±0.45), large (±0.8), very large (±1.26), and extremely large (±2.02). Rejection of the non-inferiority or non-superiority hypothesis occurs when compatibility limits do not enter the grey area. Effects with compatibility limits that end within the grey area have adequate precision but are only possibly or likely substantial. M1x, men's single sculls; W1x, women's singles sculls; M2-, men's coxless pairs; W2-, women's coxless pairs.

Between-crew differences in the effects were similar with adjustment for power and with adjustment for stroke rate and power but sometimes a little smaller when adjusting for stroke rate and power, presented in Supplementary Table 5 (adjustment for power) and Figure 4 and Supplementary Table 6 (adjustment for stroke rate and power) as standard deviations. Most effects were reduced in magnitude to the range of small to large and had inadequate precision (both substantial hypothesis were not rejected, p_+_ and p_−_ > 0.05). Between-crew differences were found in men's singles for most predictors, where precision was sufficient for the true magnitudes of the effect to be decisively substantial (rejection of the non-substantial hypotheses, p_N+_ or p_N−_ <0.05). Between-crew differences in women's singles were decisively substantial for most oar angle variables and some force and for time and velocity variables. Some force variables had decisively substantial between-crew differences in women's pairs. Predictors with adequate precision in some boat classes that were only possibly or likely substantial (one of the non-superiority or non-inferiority hypotheses was not rejected, p_N+_ or p_N−_ > 0.05) included stroke rate, time to peak force from the catch, peak force angle, and finish angle when adjusting for power, as well as peak force, rate of force development, and peak force angle when adjusting for stroke rate and power.

## Discussion

Substantial stroke-to-stroke relationships between many biomechanical variables and boat velocity have been revealed in this study of national-pathway rowers performing 2,000 m races in instrumented rowing boats, which can be used to guide technical analysis and feedback by practitioners. There was also evidence of substantial relationships for some variables after adjustment for power output and adjustment for power output and stoke rate; as such, these relationships provide potential strategies for improving boat velocity through improvement of rowing efficiency. A low risk of harm to performance (as denoted in bold in Supplementary Tables 1–3) was found for most effects before adjustment and for some effects after adjustment for power and adjustment for power and stroke rate. Practitioners can take confidence in effects with a low risk of harm to performance, as they are most unlikely to have a detrimental relationship with performance. Most variables also showed evidence of substantial individual differences in their relationship with boat velocity before adjustment for power output, and evidence of individual differences for some variables and in some boat classes was found after adjustment for power and adjustment for power and stroke rate.

In comparison to effects without adjustment, the adjustment for power resulted in large reductions in effect magnitudes across boat classes in most predictors, with many effects no longer having adequate precision, revealing the mediating effect of power output on many of the predictors assessed. The adjustment for power aimed to identify predictors that were associated with improved rowing efficiency, but the large to very large effects for stroke rate after adjustment for power highlighted the need for its supplementary adjustment with power. The additional adjustment for stroke rate with adjustment for power resulted in further reductions in effect magnitudes in many of the predictors assessed, albeit to a smaller extent than those observed with adjustment for power alone. However, the smaller reduction in effects after adjustment for stroke rate and power does not demonstrate that stroke rate has a smaller mediating effect than power output, as reductions in effect magnitudes similar to those with adjustment for power can be expected with adjustment for stroke rate alone given the strong relationship between stroke rate and power output (Hofmijster et al., 2007; Held et al., 2019). Extremely large positive effects were found in all boat classes for both power and stroke rate without adjustment, which agree with strong correlations with velocity for power (*r* = 0.44–0.67) (Coker, 2010) and stroke rate (*r* ≥ 0.66) (Martin and Bernfield, 1980; Kleshnev et al., 2001) reported in other studies, and promote the achievement of higher power outputs and stroke rates for performance improvement. In addition to the extremely large effects for power and stroke rate, their mediating effects on the relationships between most predictor variables with velocity highlights the importance of adjustment for both stroke rate and power output in the assessment of biomechanical variables in rowing.

Mean and peak force had the greatest modifying effects on velocity without adjustment for power, with extremely large positive effects observed in all boat classes. The effects for mean and peak force align with the moderate to large relationships (*r* = 0.49–0.54) between peak force and velocity observed in two elite single scullers (Coker, 2010) and likely improve velocity as a result of increased stroke power. The direction of effects for mean and peak force became negative with adjustment for power, which likely reflects faster oar angular velocities during the drive, corresponding to reduced force for a given power output per stroke as explained by the force–velocity relationship in skeletal muscle. Faster oar angular velocity may therefore benefit performance, as an optimum stroke rate for power output has not yet been identified in rowing (Held et al., 2019).

The ratio of mean to peak force during the drive provides a measure of force curve smoothness, with smoother force curves suggested to reduce within-stroke velocity fluctuations (Smith and Spinks, 1995) and boat drag (Greidanus et al., 2016). Smaller mean-to-peak force ratios associated with more successful rowers (Smith and Draper, 2006) agree with the very large negative effects for mean-to-peak force ratio found in some boat classes before adjustment for power. However, effects for the mean-to-peak force ratio variable were mostly trivial, with inadequate precision after adjustment for power and stroke rate, whereby the relationship between force curve smoothness and rowing performance appears to be mediated by power and stroke rate. Within-stroke velocity fluctuations can also be explained by power and stroke rate, as the extremely large positive effects for within-stroke velocity range before adjustment are reduced to mostly trivial effects with inadequate precision after adjustment for power and stroke rate. Associations between velocity fluctuations, stroke rate, and power have also been established (Hofmijster et al., 2007), which relate to changes in rower center of mass acceleration during the stroke (Hill and Fahrig, 2009).

The occurrence of peak force was investigated in the current study by assessment of the modifying effects on velocity of time from the catch to minimum velocity, rate of force development, time to peak force from the catch, and peak force angle. The location of peak force during the drive has been disputed in the literature, with initial studies promoting peak force to occur mid-drive due to the larger component of force application in the propulsive direction where the oar is perpendicular to the boat's long axis (Celentano et al., 1974; Martin and Bernfield, 1980). However, more recent research supports the achievement of peak force early in the drive (Millward, 1987; Kleshnev et al., 2006; Kleshnev, 2007; Coker, 2010; Warmenhoven et al., 2017; Hume, 2018), which is suggested to increase power output through increasing the area under the force–time curve, with earlier peak forces via a greater rate of force development increasing the impulse achieved (Millward, 1987; Hume, 2018), resulting in a more even distribution of power through the drive and reducing within-stroke boat velocity fluctuations (Kleshnev et al., 2006). More pronounced front-peaked force-angle curves have also been associated with rowing success in experienced female scullers, relating to increased rate of force development and the earlier location of peak force (Warmenhoven et al., 2017). The decisively substantial negative effects for time from the catch to minimum velocity and time to peak force from the catch, and the decisively substantial positive effects for rate of force development before adjustment, indicate a performance benefit for earlier peak forces. However, the reduction of effect magnitudes and loss of adequate precision in most boat classes after adjustment for power for time from the catch to minimum velocity, time to peak force from the catch, and rate of force development suggest that earlier peak forces improve velocity through increasing power output.

The increased precision in positive effects for distance per stroke with adjustment for power, in comparison to without adjustment, can be expected to reflect displacement in the calculation of velocity, whereby an increase in the distance traveled for a given power output will result in a higher velocity. Research investigating distance per stroke and stroke rate in elite rowers during 2,000 m racing found an emphasis on distance per stroke more so than stroke rate in singles, whereas pairs appeared to achieve higher stroke rates at the cost of distance per stroke (Kleshnev et al., 2001), although the results of the current study were not consistent with these findings.

Inconsistencies in the precision of effects between boat classes before adjustment for power and stroke rate make interpretation of the importance of catch and finish angles difficult. Larger negative catch and positive finish angles are understood to be advantageous, given that propulsive work is greater for a given applied force where arc angle increases (Warmenhoven et al., 2018). However, consistent modifying effects with adequate precision across boat classes were not established before adjustment for power and stroke rate, aligning with research that has also failed to establish consistent relationships between oar angle measures and boat velocity (Coker, 2010). Nevertheless, the decisively substantial effects in some boat classes for catch angle and arc angle, and the likely or possibly substantial effects in the remaining boat classes for these variables after adjustment for power and stroke rate, indicate larger catch angles to be advantageous to performance. The negative effects for catch angle likely illustrate lift forces acting on the blade at the catch, where the blade acts as a hydrofoil, increasing forward propulsion of the boat through improved mechanical efficiency (Pulman, 2005; Coker, 2010; Warmenhoven et al., 2018; Robert et al., 2019).

Catch and finish slips are a measure of the angle rowed through at either end of the stroke that does not contribute to forward propulsion of the boat. The measurement of catch and finish slips by the use of predetermined force thresholds, as those used in the current study, has been criticized for lack of individualization of the force threshold corresponding to forward boat propulsion and for measurement inaccuracies resulting from resistive forces at the blade erroneously reducing slip values (Macrossan and Macrossan, 2006). However, the extremely large negative effects observed in most boat classes without adjustment for power support the use of catch and finish slip assessment with predetermined force thresholds. Further, the reduction of effect magnitudes across boat classes for catch and finish slips after adjustment for power indicates that their effect on velocity corresponds to increased power, likely via increasing the area under the force-angle curve, and therefore propulsive work.

The between-crew differences for catch angle in men's singles after adjustment for power and stroke rate, and for most predictors in some boat classes before adjustment for power (Figure 4 and Supplementary Table 4, respectively), demonstrate the crew-specific nature of the relationships between some measures of rowing technique and boat velocity. Where a predictor has a substantial mean effect and substantial between-crew differences, both with adequate precision, we can expect a change in the predictor to be associated with a change in performance in the same direction in most rowers. However, the predictor would be best investigated on an individual basis to determine whether it is an avenue for substantial performance enhancement in each crew. The random effect solutions for crew identity present individual differences from the mean modifying effect for each crew, allowing identification of crews with greater or smaller effects for each variable. An individual approach to technical analysis using methods such as these is recommended for predictors where between-crew differences were evident, enabling individualized coaching feedback.

### Limitations

Given the assessment of predictors separately, some effects will represent underlying relationships between the predictors, whereby a change in one predictor likely contributes to changes in others. Therefore, the effects presented cannot be expected to have an additive enhancement on performance (other than those in Figure 2 with power output and those in Figure 3 with power output and stroke rate). Rather, the results present the extent to which predictors are associated with a change in velocity, informing the assessment of these variables by practitioners, with the adjustment for power and stroke identifying predictors mediated by these variables. Additionally, non-final races were included in the analyses, where crews may have implemented sub-maximal pacing strategies. However, as the current study assessed stroke-to-stroke changes in predictors, variations in predictors due to changes in pacing strategy further explain the relationships between predictors and boat velocity.

### Practical Applications

- Higher stroke rates should be targeted in racing to improve rowing performance.
- Rower force development should be prioritized as a key component of power output and boat velocity.
- The achievement of greater catch angles should be targeted in rowers and will likely improve velocity via the improved mechanical efficiency associated with lift forces at the catch.
- Relationships between some measures of rowing technique and performance are variable between rowers and are best assessed on an individual basis to determine areas of coaching focus.
- Stroke rate and power output have mediating effects on many biomechanical rowing variables, and their adjustment should be considered for future analyses of the relationships between the variables presented and velocity.

## Conclusion

The results presented suggest key areas for rowing performance improvement, such as force development, achievement of higher stroke rates, reduction of catch and finish slips, and achievement of greater catch angles. An individual approach to technical analysis and feedback is recommended, given the potentially wide between-crew differences in the effect of technique on performance without adjustment for power. The adjustment of power output and stroke rate is recommended for future research investigating the relationships between biomechanical variables and velocity in rowing due to the mediating effects of power and stroke rate observed in this study.

## Data Availability Statement

The raw data supporting the conclusions of this article will be made available by the authors on request, without undue reservation.

## Ethics Statement

The studies involving human participants were reviewed and approved by Victoria University Human Research Ethics Committee (VUHREC). The patients/participants provided their written informed consent to participate in this study.

## Author Contributions

AH, RA, RS, WH, and KB designed the study and wrote the paper. AH collected study data. WH performed statistical analyses. All authors contributed to the article and approved the submitted version.

## Conflict of Interest

The authors declare that the research was conducted in the absence of any commercial or financial relationships that could be construed as a potential conflict of interest.
