# Corrigendum: Technical Determinants of On-Water Rowing Performance

**DOI:** 10.3389/fspor.2021.681766

**Published:** 2021-04-22

**Authors:** Ana C. Holt, Robert J. Aughey, Kevin Ball, William G. Hopkins, Rodney Siegel

**Affiliations:** ^1^Institute for Health and Sport (IHeS), Victoria University, Melbourne, VIC, Australia; ^2^Sport Science Department, Victorian Institute of Sport, Melbourne, VIC, Australia; ^3^Australian Institute of Sport, Canberra, ACT, Australia

**Keywords:** technique, force curve, oar angle, race, stroke rate, power output, individual differences

In the original article, there was a mistake in Table 1 as published. Incorrect values for Power Output (W) were entered into Table 1, as the original publication includes log units instead of Watts. The corrected Table 1 appears below.

**Table 1:** Characteristics of the predictor variables in the four boat classes. Data are mean ± between-crew SD/within-crew SD^a^.

	**Single sculls**		**Coxless pairs**	
	**Men (M1x)**	**Women (W1x)**	**Men (M2-)**	**Women (W2-)**
**Time and boat-velocity variables**				
Stroke rate (strokes·min^−1^)	34.7 ± 1.7/2.0	32.8 ± 1.0/1.8	38.1 ± 0.7/1.7	35.1 ± 2.0/2.3
Within-stroke velocity range (m·s^−1^)	2.27 ± 0.20/0.13	2.14 ± 0.12/0.12	2.71 ± 0.11/0.12	2.30 ± 0.12/0.14
Time from catch to minimum velocity (s)	0.14 ± 0.03/0.04	0.12 ± 0.04/0.02	0.13 ± 0.00/0.01	0.15 ± 0.01/0.03
Distance per stroke (m)	7.97 ± 0.43/0.33	7.65 ± 0.18/0.29	7.82 ± 0.19/0.17	7.38 ± 0.34/0.28
**Gate force variables**				
Mean force (N)	261 ± 26/21	199 ± 17/16	503 ± 40/40	367 ± 34/38
Power output (W)	334 ± 33/34	223 ± 21/25	760 ± 38/92	481 ± 43/58
Peak force (N)	497 ± 68/34	371 ± 48/30	968 ± 81/65	694 ± 97/68
Rate of force development (N·s^−1^)	960 ± 190/100	760 ± 270/190	1980 ± 240/190	1450 ± 270/200
Time to peak force from the catch (s)	0.43 ± 0.07/0.04	0.39 ± 0.04/0.04	0.36 ± 0.03/0.03	0.36 ± 0.07/0.05
Mean to peak force ratio	1.90 ± 0.13/0.08	1.87 ± 0.13/0.06	1.88 ± 0.16/0.06	1.89 ± 0.13/0.08
Peak force angle (°)	−20.1 ± 5.2/4.0	−28.5 ± 6.4/4.3	−14.5 ± 3.1/3.0	−18.5 ± 4.2/2.9
**Gate angle variables**				
Catch slip (°)	7.7 ± 3.0/1.8	9.7 ± 2.8/1.8	3.7 ± 3.2/1.2	5.6 ± 3.6/2.3
Finish slip (°)	14.1 ± 3.0/1.6	18.1 ± 3.5/2.1	8.5 ± 2.9/1.0	8.5 ± 2.0/1.5
Finish angle (°)	43.5 ± 2.3/1.4	44.2 ± 3.1/1.3	33.2 ± 1.1/1.4	32.4 ± 1.7/1.2
Arc angle (°)	105.4 ± 5.2/2.5	106.0 ± 3.2/2.2	82.0 ± 2.3/1.2	80.4 ± 3.5/2.8
Catch angle (°)	−62.0 ± 5.2/1.8	−61.8 ± 2.3/1.3	−48.8 ± 2.8/1.7	−48 ± 3.8/2.0

*M1x, men's single scull; W1x, women's single scull; M2-, men's coxless pairs; W2- women's coxless pairs*.

a *Mean is the mean of the crew means, between-crew SD is the SD of the crew means, and within-crew SD is the mean of the crews' SDs across their one to the three races (~250 to ~750 strokes)*.

*Number of crews: 10, 8, 3, and 6 respectively*.

*Number of races: 17, 13, 5, and 12 respectively*.

The authors apologize for this error and state that this does not change the scientific conclusions of the article in any way. The original article has been updated.

