# Supplementary material for: Technical Determinants of On-Water Rowing Performance
Source: Front Sports Act Living. 2020 Dec 3;2:589013. doi: 10.3389/fspor.2020.589013 (PMC7739831; doi:10.3389/fspor.2020.589013)
Supplement: Supplementary file 1 [file Table_1.docx]

Supplementary Material

| **Supplementary Table 1**. Change in boat velocity for a change in predictor variables of two within-crew standard deviations without adjustment in the four boat classes. Data are mean (%), ±90% compatibility limits, with observed magnitude and p values for non-inferiority and non-superiority tests (p_–_/p_+_). | | | | |
| --- | --- | --- | --- | --- |
|  | M1x | W1x | M2- | W2- |
| **Time and velocity variables** | | | | |
| Stroke rate | **7.0, ±2.1;**  **e.large******  <0.001/>0.999 | **7.6, ±0.8;**  **e.large******  <0.001/>0.999 | **8.6, ±2.7;**  **e.large*****  0.005/0.996 | **9.0, ±1.2;**  **e.large******  <0.001/>0.999 |
| Within-stroke velocity range | **6.5, ±1.4;**  **e.large******  <0.001/>0.999 | **7.1, ±0.7;**  **e.large******  <0.001/>0.999 | 7.2, ±3.9;  e.large***  0.01/0.98 | **9.0, ±0.9;**  **e.large******  <0.001/>0.999 |
| Time from catch to minimum velocity | **-6.5, ±3.5;**  **e.large*****  0.996/0.004 | **-4.0, ±3.0;**  **v.large******  >0.999/<0.001 | -4.0, ±3.0;  v.large***  0.96/0.03 | -5.0, ±3.6;  e.large***  0.98/0.02 |
| Distance per stroke | 0.9, ±1.7;  mod  0.10/0.75 | 0.1, ±2.5;  trivial  0.39/0.44 | 2.9, ±5.7;  v.large  0.12/0.85 | 0.0, ±3.4;  trivial  0.42/0.44 |
| **Force variables** | | | | |
| Power output | **7.1, ±0.8;**  **e.large******  <0.001/>0.999 | **7.8, ±0.3;**  **e.large******  <0.001/>0.999 | **8.9, ±1.2;**  **e.large******  0.001/0.999 | **9.5, ±1.8;**  **e.large******  <0.001/>0.999 |
| Mean force | **8.2, ±1.3;**  **e.large******  <0.001/>0.999 | **9.2, ±1.0;**  **e.large******  <0.001/>0.999 | **9.1, ±2.8;**  **e.large******  0.002/0.998 | **13.3, ±5.4;**  **e.large*****  0.001/0.999 |
| Peak force | **7.4, ±1.4;**  **e.large******  <0.001/>0.999 | **9.6, ±1.5;**  **e.large******  <0.001/>0.999 | 8.1, ±4.2;  e.large***  0.007/0.99 | **13.4, ±6.5;**  **e.large******  0.003/0.997 |
| Rate of force development | **3.0, ±0.8;**  **v.large******  <0.001/>0.999 | **3.1, ±0.8;**  **v.large******  <0.001/>0.999 | 3.7, ±2.8;  v.large***  0.02/0.97 | **4.7, ±1.3;**  **e.large******  <0.001/>0.999 |
| Time to peak force from the catch | **-2.4, ±0.8;**  **large******  >0.999/<0.001 | **-3.2, ±2.3;**  **v.large******  0.999/<0.001 | -3.0, ±2.5;  v.large***  0.96/0.03 | **-4.4, ±2.0;**  **e.large******  0.998/0.001 |
| Mean to peak force ratio | **-2.5, ±0.7;**  **v.large******  >0.999/<0.001 | -1.2, ±0.9;  mod**  0.95/0.006 | -3.6, ±1.9;  v.large***  0.99/0.007 | -2.1, ±2.1;  large**  0.92/0.04 |
| Peak force angle | 0.8, ±1.0;  small**  0.03/0.81 | 0.5, ±1.4;  small  0.16/0.61 | 1.6, ±1.4;  large**  0.03/0.94 | 0.9, ±2.1;  mod  0.15/0.71 |
| **Oar angle variables** | | | | |
| Catch slip | **-2.9, ±0.8;**  **v.large******  >0.999/<0.001 | **-4.7, ±1.3;**  **e.large******  >0.999/<0.001 | **-3.6, ±1.1;**  **v.large******  0.998/0.001 | -1.0, ±8.7;  mod  0.56/0.39 |
| Finish slip | **-2.7, ±0.6;**  **v.large******  >0.999/<0.001 | **-5.0, ±1.1;**  **e.large******  >0.999/<0.001 | -3.5, ±2.7;  v.large***  0.97/0.03 | -4.8, ±2.8;  e.large***  0.99/0.005 |
| Finish angle | **-1.4, ±0.9;**  **mod*****  0.98/0.002 | -1.6, ±1.4;  large**  0.94/0.02 | 1.2, ±15.8;  mod  0.41/0.56 | -5.7, ±5.3;  e.large**  0.95/0.04 |
| Arc angle | -1.6, ±1.1;  large***  0.98/0.004 | -1.3, ±1.1;  mod**  0.93/0.01 | 1.8, ±2.6;  large  0.08/0.87 | -6.0, ±8.0;  e.large  0.89/0.09 |
| Catch angle | 0.9, ±1.2;  mod  0.05/0.80 | 0.0, ±0.9;  trivial  0.27/0.31 | -4.2, ±2.6;  e.large***  0.98/0.01 | 3.9, ±6.6;  v.large  0.12/0.85 |
| M1x, men’s single scull; W1x, women’s single scull; M2-, men’s coxless pairs; W2- women’s coxless pairs.  Number of crews: 10, 8, 3 and 6 respectively.  Number of races: 17, 13, 5, 12 respectively.  Scale of magnitudes: <0.3%, trivial; 0.3-0.9%, small; 0.9-1.6%, moderate (mod); 1.6-2.5%, large; 2.5-4.1%, very large (v.large); >4.1%, extremely large (e.large).  Reference-Bayesian likelihoods of substantial change: *possibly; **likely; ***very likely, ****most likely.  *** and **** indicate rejection of the non-superiority or non-inferiority hypothesis (p_N-_ or p_N+_ <0.05 and <0.005 respectively).  Reference-Bayesian likelihoods of trivial change: ^0^possibly; ^00^likely; ^000^very likely, ^0000^most likely.  Likelihoods are not shown for effects with inadequate precision at the 90% level (failure to reject any hypotheses: p>0.05).  Effects in **bold** have adequate precision at the 99% level (p<0.005). | | | | |
