# Supplementary material for: Technical Determinants of On-Water Rowing Performance
Source: Front Sports Act Living. 2020 Dec 3;2:589013. doi: 10.3389/fspor.2020.589013 (PMC7739831; doi:10.3389/fspor.2020.589013)
Supplement: Supplementary file 2 [file Table_2.docx]

Supplementary Material

| **Supplementary Table 2**. Change in boat velocity for a change in predictor variables of two within-crew standard deviations with adjustment for power output in the four boat classes. Data are mean (%), ±90% compatibility limits, with observed magnitude and p values for non-inferiority and non-superiority tests (p_–_/p_+_). | | | | |
| --- | --- | --- | --- | --- |
|  | M1x | W1x | M2- | W2- |
| **Time and velocity variables** | | | | |
| Stroke rate | **2.5, ±1.3;**  **v.large*****  0.002/0.99 | **2.1, ±0.8;**  **large******  <0.001/0.998 | **3.5, ±0.4;**  **v.large******  <0.001/>0.999 | **2.5, ±1.0;**  **v.large******  0.001/0.997 |
| Within-stroke velocity range | 0.4, ±0.4;  small**  0.01/0.67 | 0.0, ±0.6;  trivial  0.16/0.22 | 0.3, ±1.5;  small  0.14/0.48 | 1.1, ±0.8;  mod**  0.01/0.95 |
| Time from catch to minimum velocity | -0.6, ±0.8;  small*  0.73/0.04 | -0.4, ±0.4;  small*^0^  0.63/0.01 | -0.3, ±1.1;  small  0.55/0.09 | -0.4, ±0.5;  small*  0.75/0.04 |
| Distance per stroke | **2.7, ±1.1;**  **v.large******  <0.001/0.999 | **2.3, ±1.2;**  **large*****  0.002/0.99 | 2.1, ±2.8;  large  0.06/0.92 | 1.5, ±1.1;  v.large***  0.01/0.96 |
| **Force variables** | | | | |
| Mean force | **-3.0, ±1.4;**  **v.large******  0.998/<0.001 | **-4.1, ±1.2;**  **e.large******  0.999/<0.001 | **-4.9, ±1.4;**  **e.large*****  0.998/<0.001 | -3.7, ±2.7;  v.large***  0.94/0.01 |
| Peak force | **-2.2, ±0.9;**  **large******  0.995/<0.001 | **-2.5, ±0.7;**  **v.large******  >0.999/>0.001 | -3.8, ±2.0;  v.large***  0.98/0.009 | **-3.0, ±1.8;**  **v.large*****  0.99/0.004 |
| Rate of force development | -0.3, ±0.4;  small*^0^  0.46/0.006 | **-0.2, ±0.2;**  **trivial^00^**  0.23/0.002 | -0.1, ±0.6;  trivial  0.22/0.10 | 0.3, ±0.5;  small*^0^  0.02/0.10 |
| Time to peak force from the catch | **-0.5, ±0.3;**  **small****  0.91/<0.001 | **-0.5, ±0.2;**  **small*****  0.99/<0.001 | -0.6, ±0.5;  small**  0.79/0.04 | **-1.1, ±0.6;**  **mod*****  0.98/0.001 |
| Mean to peak force ratio | **-0.3, ±0.2;**  **small*^0^**  0.44/<0.001 | **-0.2, ±0.2;**  **trivial^00^**  0.14/0.01 | -0.3, ±0.2;  small*^0^  0.49/0.003 | -0.4, ±0.4;  small*^0^  0.65/0.09 |
| Peak force angle | **0.1, ±0.2;**  **trivial^00^**  0.002/0.12 | **-0.1, ±0.2;**  **trivial^000^**  0.02/0.004 | -0.3, ±0.4;  small*^0^  0.43/0.03 | **-0.4, ±0.3;**  **small*^0^**  0.67/0.001 |
| **Oar angle variables** | | | | |
| Catch slip | 0.1, ±0.3;  trivial^00^  0.02/0.14 | 0.2, ±0.3;  trivial^00^  0.006/0.24 | **-0.2, ±0.2;**  **trivial^00^**  0.24/0.001 | -0.2, ±0.2;  trivial^00^  0.24/0.005 |
| Finish slip | -0.1, ±0.2;  trivial^00^  0.06/0.01 | **0.1, ±0.2;**  **trivial^00^**  0.004/0.06 | 0.0, ±0.5;  trivial  0.10/0.13 | -0.3, ±0.3;  small*^0^  0.54/0.006 |
| Finish angle | **-0.4, ±0.2;**  **small****  0.84/<0.001 | -0.2, ±0.3;  trivial^0^*  0.34/0.01 | -0.3, ±0.6;  small  0.46/0.05 | -0.1, ±1.0;  trivial  0.38/0.20 |
| Arc angle | -0.3, ±0.4;  small*^0^  0.50/0.009 | 0.0, ±0.3;  trivial^00^  0.03/0.07 | 0.0, ±0.5;  trivial  0.11/0.13 | 0.2, ±1.0;  trivial  0.18/0.40 |
| Catch angle | 0.0, ±0.5;  trivial  0.14/0.15 | -0.3, ±0.4;  small*^0^  0.50/0.007 | -0.6, ±0.2;  small  0.72/0.11 | -0.2, ±0.5;  trivial  0.33/0.06 |
| M1x, men’s single scull; W1x, women’s single scull; M2-, men’s coxless pairs; W2- women’s coxless pairs.  Number of crews: 10, 8, 3 and 6 respectively.  Number of races: 17, 13, 5, 12 respectively.  Scale of magnitudes: <0.3%, trivial; 0.3-0.9%, small; 0.9-1.6%, moderate (mod); 1.6-2.5%, large; 2.5-4.1%, very large (v.large); >4.1%, extremely large (e.large).  Reference-Bayesian likelihoods of substantial change: *possibly; **likely; ***very likely, ****most likely.  *** and **** indicate rejection of the non-superiority or non-inferiority hypothesis (p_N-_ or p_N+_ <0.05 and <0.005 respectively).  Reference-Bayesian likelihoods of trivial change: ^0^possibly; ^00^likely; ^000^very likely, ^0000^most likely.  Likelihoods are not shown for effects with inadequate precision at the 90% level (failure to reject any hypotheses: p>0.05).  Effects in **bold** have adequate precision at the 99% level (p<0.005). | | | | |
