# Supplementary material for: Technical Determinants of On-Water Rowing Performance
Source: Front Sports Act Living. 2020 Dec 3;2:589013. doi: 10.3389/fspor.2020.589013 (PMC7739831; doi:10.3389/fspor.2020.589013)
Supplement: Supplementary file 3 [file Table_3.docx]

Supplementary Material

| **Supplementary Table 3**. Change in boat velocity for a change in predictor variables of two within-crew standard deviations with adjustment for stroke rate and power in the four boat classes. Data are mean (%), ±90% compatibility limits, with observed magnitude and p values for non-inferiority and non-superiority tests (p_–_/p_+_). | | | | |
| --- | --- | --- | --- | --- |
|  | M1x | W1x | M2- | W2- |
| Within-stroke velocity range | -0.4, ±0.6;  small*  0.59/0.04 | **-1.1, ±0.7;**  **mod*****  0.96/0.005 | -0.1, ±0.5;  trivial  0.23/0.08 | -0.1, ±0.6;  trivial  0.28/0.14 |
| Time from catch to minimum velocity | -0.1, ±0.8;  trivial  0.35/0.17 | -0.1, ±0.5;  trivial  0.27/0.08 | 0.3, ±0.2;  small*  <0.001/0.36 | -0.2, ±0.5;  trivial*  0.36/0.04 |
| **Force variables** | | | | |
| Mean force | **-2.2, ±1.1;**  **large*****  0.99/0.001 | **-2.4, ±1.4;**  **large*****  0.99/0.03 | **-3.2, ±1.2;**  **v.large*****  0.993/0.005 | -2.6, ±1.9;  v.large***  0.96/0.009 |
| Peak force | **-1.6, ±0.6;**  **large*****  0.99/0.001 | **-1.6, ±0.6;**  **large******  0.999/<0.001 | -1.6, ±0.6;  large****  0.996/>0.001 | -2.2, ±1.6;  large***  0.97/0.01 |
| Rate of force development | **-0.3, ±0.3;**  **small*^0^**  0.55/0.003 | **-0.3, ±0.2;**  **small*^0^**  0.47/<0.001 | -0.3, ±0.5;  small*^0^  0.51/0.03 | 0.0, ±0.4;  trivial  0.06/0.12 |
| Time to peak force from the catch | -0.2, ±0.3;  trivial^0^*  0.36/0.003 | **-0.3, ±0.2;**  **small***  0.31/<0.001 | -0.1, ±0.4;  trivial  0.14/0.05 | **-0.6, ±0.5;**  **small**  0.89/0.002 |
| Mean to peak force ratio | -0.2, ±0.5;  trivial  0.36/0.06 | -0.2, ±0.4;  trivial^0^*  0.37/0.03 | -0.0, ±0.9;  trivial  0.25/0.19 | -0.4, ±0.8;  small  0.59/0.08 |
| Peak force angle | **0.1, ±0.2;**  **trivial^000^**  0.001/0.98 | **-0.1, ±0.2;**  **trivial^00^**  0.06/0.001 | -0.3, ±0.5;  small*^0^  0.47/0.03 | **-0.5, ±0.2;**  **small****  0.90/<0.001 |
| **Oar angle variables** | | | | |
| Catch slip | 0.1, ±0.3;  trivial^00^  0.01/0.15 | **0.2, ±0.3;**  **trivial^0^**  0.003/0.32 | -0.0, ±0.3;  trivial^00^  0.08/0.04 | -0.3, ±0.2;  small*^0^  0.43/0.001 |
| Finish slip | -0.0, ±0.2;  trivial^000^  0.02/0.01 | **0.3, ±0.3;**  **small*^0^**  0.002/0.58 | 0.0, ±0.3;  trivial  0.05/0.07 | -0.3, ±0.3;  small*^0^  0.60/0.003 |
| Finish angle | -0.1, ±0.2;  trivial^00^  0.07/0.007 | **0.4, ±0.3;**  **small****  0.001/0.77 | -0.1, ±2.6;  trivial  0.43/0.32 | 0.2, ±0.9;  trivial  0.15/0.43 |
| Arc angle | 0.3, ±0.4;  small*^0^  0.02/0.46 | **1.1, ±0.3;**  **mod******  <0.001/>0.99 | **0.6, ±0.3;**  **small*****  0.002/0.97 | 1.1, ±1.1;  mod**  0.03/0.90 |
| Catch angle | -0.3, ±0.5;  small*^0^  0.52/0.02 | **-1.0, ±0.3;**  **mod******  0.998/<0.001 | -1.2, ±0.8;  mod***  0.96/0.02 | -0.9, ±0.7;  mod**  0.93/0.008 |
| M1x, men’s single scull; W1x, women’s single scull; M2-, men’s coxless pairs; W2- women’s coxless pairs.  Number of crews: 10, 8, 3 and 6 respectively.  Number of races: 17, 13, 5, 12 respectively.  Scale of magnitudes: <0.3%, trivial; 0.3-0.9%, small; 0.9-1.6%, moderate (mod); 1.6-2.5%, large; 2.5-4.1%, very large (v.large); >4.1%, extremely large (e.large).  Reference-Bayesian likelihoods of substantial change: *possibly; **likely; ***very likely, ****most likely.  *** and **** indicate rejection of the non-superiority or non-inferiority hypothesis (p_N-_ or p_N+_ <0.05 and <0.005 respectively).  Reference-Bayesian likelihoods of trivial change: ^0^possibly; ^00^likely; ^000^very likely, ^0000^most likely.  Likelihoods are not shown for effects with inadequate precision at the 90% level (failure to reject any hypotheses: p>0.05).  Effects in **bold** have adequate precision at the 99% level (p<0.005). | | | | |
