# Supplementary material for: Technical Determinants of On-Water Rowing Performance
Source: Front Sports Act Living. 2020 Dec 3;2:589013. doi: 10.3389/fspor.2020.589013 (PMC7739831; doi:10.3389/fspor.2020.589013)
Supplement: Supplementary file 4 [file Table_4.docx]

Supplementary Material

| **Supplementary Table 4**. Differences between crews in the effects of the technical variables shown in Supplementary Table 1 without adjustment in the four boat classes. Data are SD (%), ±90% compatibility limits (approximate), with observed magnitude and p values for non-inferiority and non-superiority tests (p_–_/p_+_). | | | | |
| --- | --- | --- | --- | --- |
|  | M1x | W1x | M2- | W2- |
| **Time and velocity variables** | | | | |
| Stroke rate | 3.4, ±1.5;  e.large***  0.02/0.98 | 1.1, ±0.8;  large  0.05/0.94 | 1.5, ±1.8;  v.large  0.16/0.83 | 1.3, ±1.1;  v.large  0.06/0.93 |
| Within-stroke velocity range | 2.2, ±1.0;  e.large***  0.02/0.98 | 1.0, ±0.7;  large**  0.05/0.95 | 2.1, ±2.6;  e.large  0.17/0.83 | 1.0, ±0.9;  large  0.07/0.92 |
| Time from catch to minimum velocity | 6.4, ±3.0;  e.large***  0.02/0.98 | 1.9, ±1.1;  v.large***  0.04/0.96 | 1.8, ±2.3;  v.large  0.18/0.82 | 4.6, ±4.0;  e.large  0.06/0.94 |
| Distance per stroke | 2.7, ±1.3;  e.large***  0.02/0.98 | 3.7, ±2.0;  e.large***  0.03/0.97 | 3.2, ±4.0;  e.large  0.16/0.84 | 4.0, ±3.5;  e.large  0.06/0.94 |
| **Force variables** | | | | |
| Mean force | **2.1, ±0.7;**  **e.large******  0.002/0.998 | 1.4, ±0.5;  v.large***  0.008/0.99 | 1.9, ±2.0;  v.large  0.11/0.89 | 6.1, ±3.5;  e.large***  0.04/0.97 |
| Power output | 1.2, ±0.6;  large***  0.02/0.97 | 0.4, ±0.3;  small**  0.04/0.90 | 0.6, ±0.8;  mod  0.18/0.79 | 2.0, ±1.7;  v.large  0.06/0.94 |
| Peak force | **2.3, ±0.7;**  **e.large******  0.003/0.997 | 2.1, ±0.8;  e.large***  0.007/0.99 | 3.0, ±3.1;  e.large  0.10/0.90 | 7.1, ±4.9;  e.large***  0.05/0.96 |
| Rate of force development | **1.9, ±0.6;**  **large******  0.002/0.997 | 1.6, ±0.6;  large***  0.008/0.99 | 3.1, ±3.0;  e.large  0.08/0.92 | 2.3, ±0.9;  e.large***  0.03/0.97 |
| Time to peak force from the catch | **2.0, ±0.7;**  **v.large*****  0.005/0.996 | 2.8, ±1.2;  e.large***  0.02/0.98 | 2.3, ±2.8;  e.large  0.15/0.85 | 3.9, ±1.7;  e.large***  0.01/0.99 |
| Mean to peak force ratio | **1.9, ±0.6;**  **v.large******  0.002/0.997 | 2.1, ±0.8;  e.large***  0.006/0.99 | 2.2, ±2.2;  e.large  0.10/0.90 | 4.1, ±1.8;  e.large***  0.02/0.99 |
| Peak force angle | **2.5, ±0.8;**  **e.large******  0.003/0.997 | 3.2, ±1.2;  e.large***  0.01/0.99 | 1.3, ±1.6;  v.large  0.16/0.83 | 3.7, ±1.9;  e.large***  0.03/0.97 |
| **Oar angle variables** | | | | |
| Catch slip | **2.0, ±0.7;**  **v.large******  0.004/0.996 | 3.0, ±1.3;  e.large***  0.02/0.99 | 1.3, ±1.3;  v.large  0.10/0.90 | 14.6, ±14.1;  e.large  0.07/0.93 |
| Finish slip | **1.6, ±0.5;**  **v.large******  0.003/0.997 | 2.5, ±1.2;  e.large***  0.02/0.98 | 2.6, ±3.1;  e.large  0.14/0.86 | 5.5, ±2.6;  e.large***  0.02/0.98 |
| Finish angle | **2.3, ±0.8;**  **e.large******  0.004/0.996 | 3.1, ±1.3;  e.large***  0.01/0.99 | 13.7, ±17.4;  e.large  0.15/0.85 | 9.9, ±7.0;  e.large***  0.05/0.96 |
| Arc angle | **2.8, ±0.9;**  **e.large******  0.003/0.997 | 2.5, ±1.0;  e.large***  0.008/0.99 | 2.7, ±2.9;  e.large  0.11/0.89 | 14.3, ±13.7;  e.large  0.07/0.93 |
| Catch angle | **3.1, ±1.0;**  **e.large******  0.003/0.997 | 2.0, ±0.8;  v.large***  0.008/0.99 | 2.8, ±3.1;  e.large  0.12/0.88 | 10.6, ±10.0;  e.large  0.07/0.93 |
| M1x, men’s single scull; W1x, women’s single scull; M2-, men’s coxless pairs; W2- women’s coxless pairs.  Number of crews: 10, 8, 3 and 6 respectively.  Number of races: 17, 13, 5, 12 respectively.  Scale of magnitudes: <0.15%, trivial; 0.15-0.45%, small; 0.45-0.8%, moderate (mod); 0.8-1.26%, large; 1.26-2.02%, very large (v.large); >2.02%, extremely large (e.large).  Reference-Bayesian likelihoods of substantial change: *possibly; **likely; ***very likely, ****most likely.  *** and **** indicate rejection of the non-superiority or non-inferiority hypothesis (p_N-_ or p_N+_ <0.05 and <0.005 respectively).  Likelihoods are not shown for effects with inadequate precision at the 90% level (failure to reject any hypotheses: p>0.05).  Effects in **bold** have adequate precision at the 99% level (p<0.005). | | | | |
