# Supplementary material for: Technical Determinants of On-Water Rowing Performance
Source: Front Sports Act Living. 2020 Dec 3;2:589013. doi: 10.3389/fspor.2020.589013 (PMC7739831; doi:10.3389/fspor.2020.589013)
Supplement: Supplementary file 6 [file Table_6.docx]

Supplementary Material

| **Supplementary Table 6**. Differences between crews in the effects of the technical variables shown in Supplementary Table 3 with adjustment for stroke rate and power in the four boat classes. Data are SD (%), ±90% compatibility limits (approximate), with observed magnitude and p values for non-inferiority and non-superiority tests (p_–_/p_+_). | | | | |
| --- | --- | --- | --- | --- |
|  | M1x | W1x | M2- | W2- |
| Within-stroke velocity range | 0.9, ±0.8;  large  0.05/0.94 | 1.0, ±0.9;  large  0.06/0.93 | 0.1, ±0.5;  trivial  0.40/0.48-1 | 0.7, ±0.8;  mod  0.10/0.88 |
| Time from catch to minimum velocity | 1.2, ±1.1;  large  0.08/0.91 | 0.7, ±0.6;  mod  0.07/0.90 | 0.2, ±0.4;  small  0.30/0.54 | 0.5, ±0.5;  mod  0.08/0.88 |
| **Force variables** | | | | |
| Mean force | 1.9, ±0.7;  v.large***  0.009/0.99 | 1.9, ±0.9;  v.large***  0.03/0.97 | 0.5, ±0.9;  mod  0.32/0.62 | 2.3, ±1.4;  e.large***  0.04/0.96 |
| Peak force | 1.5, ±0.6;  v.large***  0.01/0.99 | 0.8, ±0.6;  large**  0.04/0.93 | 1.0, ± 1.3;  large  0.18/0.80 | 2.1, ±1.0;  v.large  0.02/0.98 |
| Rate of force development | 0.8, ±0.4;  large***  0.006/0.99 | 0.4, ±0.2;  small**  0.02/0.92 | 0.4, ±0.5;  small  0.17/0.76 | 0.6, ±0.5;  mod**  0.04/0.94 |
| Time to peak force from the catch | 0.9, ±0.4;  large***  0.02/0.98 | 0.3, ±0.3;  small  0.05/0.87 | 0.5, ±0.6;  mod  0.17/0.79 | 1.1, ±0.6;  large***  0.03/0.96 |
| Mean to peak force ratio | 0.6, ±0.2;  mod***  0.007/0.99 | 0.4, ±0.2;  small***  0.01/0.96 | 0.3, ±0.5;  small  0.18/0.72 | 0.7, ±0.4;  large***  0.03/0.95 |
| Peak force angle | 0.4, ±0.2;  small***  0.01/0.96 | 0.3, ±0.3;  small**  0.04/0.87 | 0.5, ±0.5;  mod  0.10/0.85 | 0.3, ±0.3;  small  0.07/0.83 |
| **Oar angle variables** | | | | |
| Catch slip | 0.7, ±0.2;  mod***  0.006/0.99 | 0.5, ±0.2;  mod***  0.02/0.97 | 0.3, ±0.4;  small  0.13/0.72 | 0.3, ±0.3;  small  0.10/0.76 |
| Finish slip | 0.5, ±0.2;  mod***  0.01/0.97 | 0.5, ±0.5;  mod  0.06/0.91 | 0.2, ±0.4;  small  0.17/0.67 | 0.5, ±0.4;  mod  0.06/0.89 |
| Finish angle | 0.5, ±0.3;  mod**  0.03/0.94 | 0.7, ±0.3;  mod  0.02/0.97 | 1.8, ±2.4;  large  0.21/0.79 | 1.4 ±1.4;  v.large  0.08/0.91 |
| Arc angle | 1.1, ±0.4;  large***  0.005/0.99 | 0.5, ±0.3;  mod***  0.02/0.96 | 0.2, ±0.3;  small  0.13/0.63 | 1.5, ±1.8;  v.large  0.13/0.86 |
| Catch angle | 1.1, ±0.4;  large***  0.007/0.99 | 0.6, ±0.3;  mod***  0.02/0.96 | 0.6, ±0.9;  mod  0.24/0.74 | 1.1, ±1.0;  large  0.07/0.93 |
| M1x, men’s single scull; W1x, women’s single scull; M2-, men’s coxless pairs; W2- women’s coxless pairs.  Number of crews: 10, 8, 3 and 6 respectively.  Number of races: 17, 13, 5, 12 respectively.  Scale of magnitudes: <0.15%, trivial; 0.15-0.45%, small; 0.45-0.8%, moderate (mod); 0.8-1.26%, large; 1.26-2.02%, very large (v.large); >2.02%, extremely large (e.large).  Reference-Bayesian likelihoods of substantial change: *possibly; **likely; ***very likely, ****most likely.  *** and **** indicate rejection of the non-superiority or non-inferiority hypothesis (p_N-_ or p_N+_ <0.05 and <0.005 respectively).  Likelihoods are not shown for effects with inadequate precision at the 90% level (failure to reject any hypotheses: p>0.05).  Effects in **bold** have adequate precision at the 99% level (p<0.005). | | | | |
